# Characteristics of retracted biomedical research papers from Latin American institutions

**DOI:** 10.11606/s1518-8787.2025059006328

**Published:** 2025-10-17

**Authors:** Jorge Andres Ramos-Castaneda, Cristina Candal-Pedreira, Mónica Pérez-Ríos, Ana Teijeiro, Alberto Ruano-Ravina, Guadalupe García

**Affiliations:** I Universidad Antonio Nariño. Grupo de investigación Innovación y Cuidado. Facultad de Enfermería. Neiva, Colombia; II Universidade de Santiago de Compostela. Medicina Preventiva y Salud Pública. Santiago de Compostela. Galicia, Spain; III Centro de Investigación Biomédica en Red en Epidemiología y Salud Pública. Madrid, Spain; IV Instituto de Investigación Sanitaria de Santiago de Compostela. Santiago de Compostela, Galicia, Spain

**Keywords:** Fraud, Scientific Misconduct, Ethics, Latin America

## Abstract

**OBJECTIVE:**

To characterize retractions of biomedical research papers that had a least one author affiliated with a Latin American (LATAM) institution.

**METHODS:**

We conducted a cross-sectional study of retracted research papers published in scientific journals focusing on the field of biomedical research and identified by means of the Retraction Watch database. The retracted articles identified were required to have at least one author whose institutional affiliation was in a LATAM country. Data were collected on the authors’ countries and institutional affiliations, the reason for retraction, dates of publication and retraction, indexing, journal quartile and impact factor. Reasons for retraction were categorized into three major groups, i.e., scientific misconduct, error, and not specified.

**RESULTS:**

According to Retraction Watch, 181 papers were retracted across 1987–2024 which fulfilled the inclusion criteria. Guatemala, Bolivia, Peru, Panama, Ecuador, Colombia, and Argentina were the countries that had a retraction rate above 1 per 10 thousand papers throughout the study period. The principal reason for retraction was scientific misconduct (63.0%) followed by honest error (21.5%). The main causes of retraction due to scientific misconduct were ethical and legal problems (33.1%), followed by fabrication/falsification (20.2%).

**CONCLUSION:**

The number of retractions in some LATAM countries, mainly due to scientific misconduct, highlights the need to strengthen ethical practices in research. Future initiatives should focus on developing and evaluating effective strategies to prevent misconduct and promote scientific integrity.

## INTRODUCTION

Retraction is defined as the method whereby science self-corrects itself^1^. This serves as a warning to the scientific community that the results and conclusions of a research paper in question are erroneous or fraudulent, and therefore invalid. The two main reasons that require a paper to be retracted are honest error and scientific misconduct. Honest error covers errors committed not intentionally by authors, such as errors in analysis, tables or figures which require a research paper to be retracted. Additionally, a journal may erroneously publish a duplicate scientific paper, thereby requiring retraction of one of the duplicated papers. For its part, misconduct encompasses a wide range of practices carried out intentionally, which undermines the principles of scientific integrity. The forms known as classic or traditional scientific misconduct are fabrication, falsification and plagiarism, though other practices are also deemed to be misconduct, such as duplication, authorship problems, or purposeful failure to disclose conflicts of interest, among others^2^.

Analysis of such retractions acts as an indicator for studies seeking to analyze scientific misconduct. Previous studies have used this method to ascertain the situation of scientific misconduct, both overall^3^ and by area of knowledge^4-6^ or authors’ country^3,7-9^. Hence, Fang et al.^3^ reported, in 2012, that the USA registered the highest frequency of papers retracted for fraud or suspicion of fraud, while China and Canada were the countries with the highest frequencies of plagiarism and duplication, respectively. Furthermore, Amos concluded that Italy had the highest ratio of retractions for plagiarism, whereas Finland ranked high in terms of duplication^10^. The results of studies based on analysis of retractions thus reveal differences in the most common types of scientific misconduct by country. Another recent study that analyzed retractions in Europe also identified differences based on country of affiliation^11^.

In South America, four studies have been conducted which analyze retractions at a national level. Three of these studies were undertaken in Brazil, with all agreeing on the fact that the most frequent type of scientific misconduct in that country was duplication/plagiarism^7,8,12^. The fourth study analyzed retractions indexed in Latin America (LATAM) and Caribbean databases until 2014, and once again reported plagiarism as being the leading type of scientific misconduct in the papers analyzed^13^. To our knowledge, however, no study has yet carried out an overall analysis of retracted papers in LATAM countries. The study of retractions in a given country or continent can be useful for indirectly establishing the frequency of misconduct, and for fostering scientific misconduct prevention and detection mechanisms adapted to a specific geographical context. This study therefore sought to quantify and characterize retractions of biomedical research papers that had at least one author affiliated with a LATAM institution.

## METHODS

### Study Design

We conducted a cross-sectional study of research papers published in scientific journals categorized in the "Basic Life Sciences (BLS)" and/or "Health Sciences (HSC)" field, which had been retracted. Retracted papers were required to have at least one author whose institutional affiliation was in a LATAM country. The types of articles included were research papers, systematic reviews/meta-analyses, conference abstracts, and others (case reports, comments/editorials).

We included all papers retracted between 1987, the year of the first retraction that fulfilled the inclusion criteria, and 2024, using the Retraction Watch database to identify articles that met the established criteria. The most recent date of access to the database was September 23^rd^, 2024.

Retraction Watch is a publicly accessible database that collects retracted, corrected and ‘expression of concern’ scientific publications, irrespective of language or the journal of publication. As of September 2024, the database contained more than 56 thousand retracted publications. To include retractions, Retraction Watch employs a comprehensive approach. In addition to traditional scientific databases such as the United States National Library of Medicine (PubMed) and Web of Science, it performs daily manual searches using keywords such as ‘retraction’, ‘withdrawn’ or ‘retracted paper’. These searches extend to publisher websites, social media and researcher comments. Additionally, they rely on institutional research reports and national or local scientific integrity offices to expand coverage of retractions that are not accessible in mainstream databases. Furthermore, they collaborate with platforms such as PubPeer and experts. Retraction Watch collects information on the reason for retraction by manually verifying retraction notes.

The countries included in the analysis as LATAM were Argentina, Bolivia, Chile, Colombia, Costa Rica, Cuba, Ecuador, El Salvador, Granada, Guatemala, Guyana, French Guiana, Haiti, Honduras, Jamaica, Mexico, Nicaragua, Panama, Paraguay, Peru, Puerto Rico, Dominican Republic, Surinam, Uruguay, and Venezuela. Brazil was excluded from this analysis as the working group had already analyzed the retractions in Brazil^12^.

### Variables and Data Source

For each of the retracted articles identified, we collected data from Retraction Watch on title, type of publication, number of authors, country and institutional affiliation of each of the authors, scientific journal, country of principal author's affiliation (considered as first, last or corresponding author of the retracted paper), date of publication, and date of retraction. The information on indexing, journal quartile and impact factor were obtained from Journal Citation Reports (JCR). We downloaded the full text of all retracted articles included to review the conflict of interest and acknowledgement of funding declarations. Citations to the original papers until October 13^th^, 2024, were collected using Google Scholar.

We identified the reason for retraction based on a search of Retraction Watch and a review of retraction notes. Reasons for retraction were then categorized into three major groups, namely, scientific misconduct, error, and not specified. For the categorization of retraction reasons, we drew upon international guidelines on scientific integrity and various practices classified as misconduct or unacceptable/questionable conduct (including sources such as the International Committee of Medical Journal Editors—ICMJE, All European Academies—ALLEA, and the US Office of Research Integrity—US ORI), in addition to previously published studies on the subject. In this analysis, we address both practices traditionally defined as scientific misconduct—namely fabrication, falsification, and plagiarism—as well as other actions commonly labeled as questionable or unacceptable. It is important to clarify that, in this study, no distinction is made between these questionable/unacceptable practices and scientific misconduct, as we classify all under the term "scientific misconduct."

Papers retracted due to scientific misconduct were classified into plagiarism, duplication, fabrication or falsification, unreliable results, or data and ethical/legal concerns. The categorization of scientific misconduct used for this study has been previously used and is described in detail in previous papers^12,14^. Where any article retracted due to scientific misconduct had more than one classification, a specific reason was designated as the principal reason by applying a scale used in previous studies with similar objectives undertaken in different geographical settings^12,14^. Error-based retractions were due to an honest error on the part of the authors or journal, and retractions classified as "not specified" were those where the reason for retraction was not specified either in Retraction Watch nor in the retraction note.

### Statistical Analysis

We performed a descriptive analysis using absolute and relative frequencies for the categorical variables. The proportion of each of the variables of interest was determined by taking into account the total number of articles retracted and the reason for retraction (misconduct, error or not specified). We also calculated the annual retraction rate, with a breakdown by the number of papers in each country and overall. To calculate the number of papers in each country, we took into account the number of articles published per year in which one or more of the authors had a LATAM country in their institutional affiliation. This information was extracted from Web of Science.

A network analysis was performed to identify collaborations on retracted articles between a LATAM country and the rest of the world, using the Fruchterman Reingold algorithm.

We conducted an analysis of the citations received by the retracted articles included. Initially, we described the distribution of citations according to the quartile of the journal of publication, constructing a box plot. Subsequently, we analyzed the citations according to the cause of retraction.

Statistical analyses were performed using the RStudio 4.2.2. program^15^, and network diagrams were constructed with the igraph library version: 1.5.0^16^.

## RESULTS

Using the Retraction Watch database, a total of 181 retracted papers were identified, which had been published across the period 1987–2024 and listed authors whose institutional affiliation was from a LATAM country. Most of the retracted papers were original research papers (82.3%), and the main author (first, last or corresponding author) was affiliated with an institution in a LATAM country in 74.6% of cases; 74.0% of the articles retracted were published in a journal indexed in Journal Citation Reports (JCR), and half were published in journals ranked in Q1 (32.2%) or Q2 (23.7%) of their category (Table 1).

**Table 1:** Main characteristics of retracted papers, by authors affiliated with Latin American institutions, overall and by reason for retraction.

		Overall		Error		Scientific misconduct		Not specified	
		n	%	n	%	n	%	n	%
OVERALL		181	100	39	21.5	114	63.0	28	15.5
Type of publication									
	Research article	149	82.3	33	84.6	96	84.2	20	71.4
	Review/meta-analysis	21	11.6	5	12.8	10	8.8	6	21.4
	Conference abstract	6	3.3	0	0	5	4.4	1	3.6
	Other*	5	2.8	1	2.6	3	2.6	1	3.6
Number of authors									
	1–3	41	22.7	9	23.1	26	22.8	6	21.4
	4–8	90	49.7	24	61.5	52	45.6	14	50.0
	More than 8	50	27.6	6	15.4	36	31.6	8	28.6
Number of affiliation countries									
	1	77	42.5	18	46.2	47	41.2	12	42.9
	2	41	22.7	14	35.9	19	16.7	8	28.6
	3	31	17.1	3	7.7	23	20.2	5	17.9
	4 or more	32	4.4	4	10.3	25	21.9	3	10.7
Author's country									
	Latin America: first, last, and/or corresponding author	135	74.6	32	82.1	79	69.3	24	85.7
	Non Latin America: first, last, and/or corresponding author	46	25.4	7	17.9	35	30.7	4	14.3
Declared conflicts of interest^+^									
	Does not contain a COI statement	64	45.4	10	31.3	45	48.9	9	52.9
	Contain a COI statement	77	54.6	22	68.7	47	51.1	8	47.1
	Conflicts with the pharmaceutical industry	8	10.4	1	4.5	6	12.8	1	12.5
Declared funding|									
	Does not contain a funding statement	48	34.5	5	14.7	34	38.2	9	56.3
	Contain a funding statement	91	65.5	29	85.3	55	61.8	7	43.7
	Declared that funding was received	80	87.9	26	89.7	47	85.4	7	100
	Declared that funding was not received	11	12.1	3	10.3	8	14.6	0	0
JCR quartile									
	Not indexed in JCR	47	26.0	5	12.8	37	32.4	5	20.8
Impact factor, median (RI)		3.0 (2.2–4.8)		3.9 (2.6–6.1)		2.7 (2.0–3.8)		3.2 (2.0–6.8)	
	Q1	57	32.2	18	46.1	29	25.4	10	41.7
	Q2	42	23.7	12	30.8	26	22.8	4	16.7
	Q3	19	10.7	4	10.2	14	12.2	1	4.2
	Q4	12	6.8	0	0	8	7.0	4	16.7

* Other: Case report (n = 2); Comments/Editorial (n = 3). COI: conflicts of interest; JCR: Journal Citations Reports. + In 44 documents the full text could not be read to identify the statement.

| 42 documents could not be read to identify the statement.

From among all the retracted articles, 16 LATAM countries were identified as appearing in some affiliation of the authors, with Mexico (30.9%), Argentina (22.7%), Colombia (16.6%), and Chile (12.2%) being the countries with the highest proportion. Within these countries, retraction for scientific misconduct occurred in 55.4% of Mexican, 63.2% of Argentinian, 66.7% of Colombian and 52.4% of Chilean retracted articles. Mexico (n = 8), Argentina (n = 8), and Colombia (n = 11) were the countries with the highest number of retracted papers due to fabrication/falsification, duplication, and ethical/legal issues, respectively.

**Figure 1:**
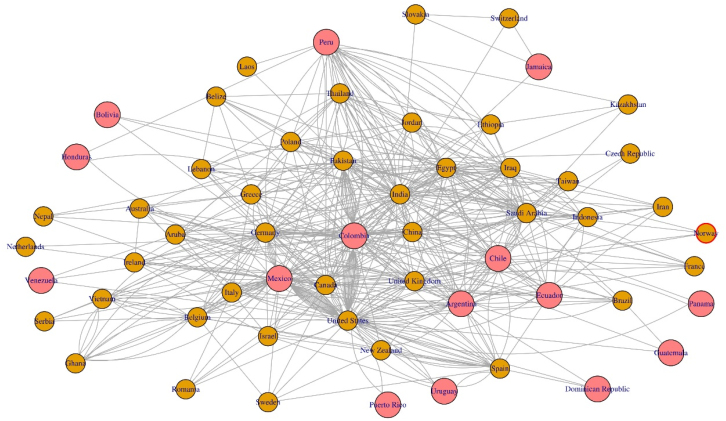
Network analysis of existing collaborations on retracted papers between Latin American institutions and other countries.

Countries appearing less frequently were Peru, Venezuela, Uruguay, Panama, Ecuador, Dominican Republic, Puerto Rico, Jamaica, Guatemala, Honduras, Cuba, and Bolivia. In a network diagram, collaborations were identified between LATAM and other countries, such as the USA (n = 44), India (n = 16), Pakistan (n = 14), China (n = 11), United Kingdom (n = 13), Spain (n = 11), Canada (n = 9), Greece (n = 4), and Israel (n = 4) (Figure 1).

**Figure 2:**
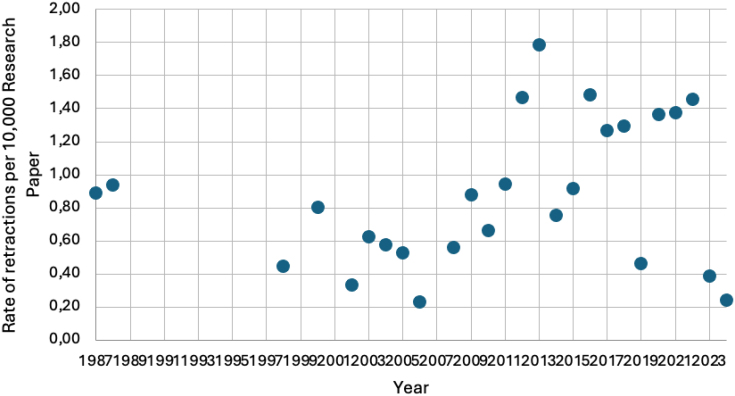
Rate of retractions per 10,000 research papers of Latin American institutions authors from 1987 to 2024.

**Figure 3:**
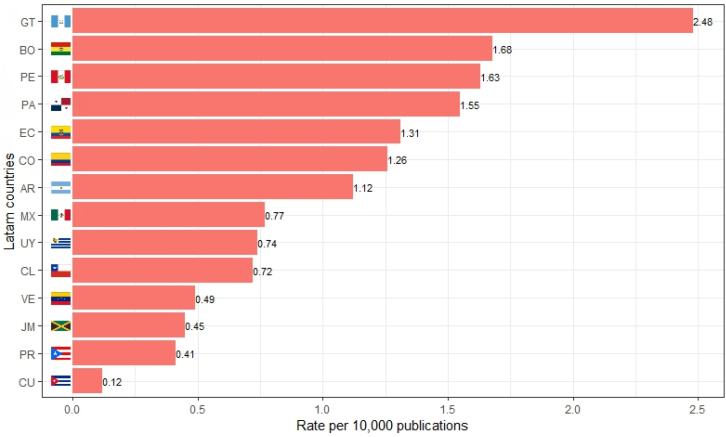
Rate of retractions per 10,000 papers for each Latin American institutions country from 1987 through 2024.

In the period from 1987 through 2010, 0.60 per 10 thousand papers were retracted in LATAM, with this figure rising to 1 per 10 thousand across the period 2011–2024. Guatemala, Bolivia, Peru, Panama, Ecuador, Colombia, and Argentina were the countries that registered a retraction rate of over 1 per 10 thousand papers throughout the study period (Figures 2 and 3).

### Reason for Retraction

The principal reason for retraction was scientific misconduct (63.0%), followed by honest error (21.5%), and in 15.5% of papers no reason for retraction could be identified. The principal cause of retraction due to scientific misconduct was ethical or legal reasons (33.1%), followed by fabrication/falsification (20.2%) (Table 2).

**Table 2:** Description of the reasons for retraction in retracted papers by authors affiliated with Latin American institutions.

		Overall	
		n	%
Error		39	21.5
Scientific misconduct		114	63.0%
	Ethical/legal issues	41	33.1
	Fabrication/falsification	25	20.2
	Duplication	22	17.7
	Unreliable data/results	19	15.3
	Plagiarism	17	13.7
Reason not specified		28	15.5
Total		181	

In general, there were no great differences in the characteristics of retracted papers, when analyzed by reason for retraction (Table 1). That said, however, 32.4% of papers retracted due to scientific misconduct were observed to have been published in journals not indexed in JCR versus 12.8% of papers retracted due to error. Furthermore, 48.9% of papers retracted due to scientific misconduct did not contain a conflict of interest declaration, as compared with 31.3% of those retracted due to error. In terms of acknowledgement of funding declarations, only 61.8% of papers retracted due to scientific misconduct contained this versus 85.3% of papers retracted due to honest error (Table 1).

### Citation Analysis

The 181 retracted papers included were cited a total of 7,036 times. It was observed that the articles from journals Q1 and Q2 had a median number of citations of 29 and 20.5 respectively (Suppl. Mat. 1, panel A), which was higher than that of articles published in Q3 and Q4 journals. In addition, no difference was identified in the median number of citations depending on the reason for retraction (scientific misconduct: 13 versus error: 12) (Suppl. Mat. 1, panel B).

## DISCUSSION

The results of this study highlight a number of issues. Firstly, scientific misconduct is the principal reason for retraction in LATAM countries, a finding in line with the scientific evidence available for other regions. Secondly, the retraction rate has increased in recent years in these countries. Thirdly, among the causes of retraction due to scientific misconduct, the main ones are ethical and legal problems, followed by fabrication/falsification, which together account the 50% of the retractions due to scientific misconduct. Lastly, there are important differences between the countries analyzed in terms of retraction percentages, with no apparent correlation between retraction and the intensity of scientific production in these countries.

Retractions are more common in the medical and health sciences field, and few studies have evaluated retractions of articles published by authors with affiliation in a LATAM country. One study specifically evaluated articles retracted in institutions in Brazil^8^, another compared retractions in Portugal with Brazil^12^, and the most recent study by Herrera-Añazco et al.^17^ included retractions between 2003 and 2022 from all the LATAM countries. Our study ascertained that the principal causes of retraction due to scientific misconduct in LATAM are ethical or legal problems, and, among these, authorship problems are preeminent (data not shown). These results are in variance with the main reasons for retraction identified in other countries and even in LATAM countries themselves. For instance, a number of authors have reported that the reason for retraction of research papers in Brazil is principally plagiarism^8,13,17^, one of the three most serious and frequent behaviors according to the US Office of Research Integrity (fabrication, falsification and plagiarism)^3,18^.

As compared with fabrication, falsification and plagiarism, problems of authorship are regarded as being of slight relevance, even though they are frequent in collaborative scientific circles^19^ and may arise in the form of guest/gift authorship or ghost authorship. It can be argued that one of the underlying reasons for the high frequency of authorship problems in LATAM countries is a lack of awareness and adherence by researchers in these countries to the authorship criteria set forth by the International Committee of Medical Journal Editors^20^, plus the fact that there is evidence to show that papers which undergo these types of retractions are mainly found in biomedical research^19^. Guest/gift authorship could be related with the pressure to publish that prevails in the academic world, where the more papers a researcher publishes, the more his/her prestige and fundraising ability grows. Even so, it should be noted that the system of evaluating researchers seems to be gradually changing. The Declaration on Research Assessment (DORA)^21^ and Coalition for Advancing Research Assessment (CoARA)^22^ are two international initiatives that seek to shift the current quantitative system toward a more qualitative system, where what really counts is a researcher's impact on his/her area of knowledge, rather than the number of papers he/she has published. To this end, DORA and CoARA reject the use of inappropriate institutional rankings and metrics.

Different bodies (research funding agencies and universities) are adopting the DORA and CoARA recommendations. For instance, the University of Utrecht has recently decided not to take part in the 2024 Times Higher Education World University Ranking, which depends in great measure on the number of papers published by researchers affiliated with a given institution^23^. Another example is Spain's Carlos III Institute of Health, which, in its latest call for leading biomedical research projects at a national level, laid down that research applicants would have to choose their ten most relevant and impactful papers, and that these, instead of their complete curriculum, would then be evaluated. Funding agencies and researchers’ institutions can impose measures that would reduce the pressure to publish and, by extension, scientific misconduct. To our knowledge, no LATAM institution has followed in the steps of the abovementioned institutions.

Therefore, it is important for scientific, academic, and governmental organizations in LATAM to work together to ensure compliance with recommendations to guarantee scientific integrity. In recent years, an increase in the number of publications and retracted articles has been observed in LATAM countries^17,24^. Additionally, the decrease in economic income in LATAM countries has an impact on the financing of research projects, and the pressure on researchers could influence non-ethical scientific practices^25^.

In the analysis of collaboration between countries, mention should be made of the relations that exist between Colombia, USA and Pakistan. A 6.6% of the Colombian articles retracted were the product of collaboration with an author from Pakistan, and all were retracted due to scientific misconduct, with authorship problems being the specific reason for retraction. Additionally, 90% of these articles listed an author from Colombia in their affiliations and all were published in the journal "Cureus" by a single author who had had 15 papers retracted^26^. Moreover, these papers had authors and researchers from Greece, USA, Canada, United Kingdom, India, and China.

Insofar as these results are concerned, it is noteworthy that almost half of the papers retracted had been published in journals ranked in the first and second quartile of their category. It is likewise noteworthy that the type of paper most frequently retracted was the original research paper. This is relevant, since these types of papers generally enjoy far greater visibility vis-à-vis the scientific community, even after retraction. Previous research has shown that retraction would not appear to have an effect on the citations received, thereby ensuring that erroneous or fraudulent information endures in the scientific literature^27-30^. Yet it should be noted that, if the characteristics of the journal of publication are analyzed by reference to the reason for retraction, the results change. LATAM papers retracted by reason of scientific misconduct are more frequently published in journals not indexed in JCR and thus subject to less scrutiny and visibility. This could be accounted for by the fact that non-indexed journals may have fewer resources for reviewing and monitoring the papers that they publish, thereby rendering these processes less rigorous. Another explanation could be that these journals do not have clear ethical policies.

In health and medical research, it is important to ensure scientific integrity because the results could have an impact on healthcare^17^. Health interventions at individual, collective and population levels are formulated based on scientific evidence. These interventions represent economic efforts for the health systems. Therefore, publications with non-ethical scientific practice can have consequences at the public health level. In this regard, it is important for all stakeholders to take coordinated actions in order to prevent scientific misconduct. Academic and scientific institutions should promote ethical education for good scientific conduct. In this context, some institutions have implemented the recommendations made by the Committee on Publication Ethics (COPE) in this regard by establishing research integrity offices and training programmes in ethics^31^. At the European level, for instance, several grants have been aimed at providing guidance to institutions on the ethics training of researchers^32^. Furthermore, it is recommended that institutions enhance transparency regarding the management of investigations of scientific misconduct, given the potential for conflicts of interest in these cases. Institutions should also facilitate a transformation in the researcher assessment system with the objective of alleviating the pressure to publish, thereby reducing the incidence of unethical behavior driven by this. Additionally, all stakeholders should establish safe channels for reporting possible scientific misconduct and ensure that there are consequences for researchers who engage in non-ethical practices.

This study has a series of advantages. First, having used such an exhaustive database as that belonging to Retraction Watch means that losses of retracted papers will likely be minimal, though there may be papers that were not located. A further advantage lies in having the analysis with a breakdown by country, something that makes it easier to evaluate possible differences between researchers according to their location. Finally, a relevant advantage is having considered the first, last or corresponding author because this enables more accurate ascertainment of the role of LATAM authors in cases where these are the principal investigators.

This study has certain limitations. We used a broad definition of scientific misconduct that includes not only fabrication, falsification, and plagiarism, but also questionable and unacceptable practices. This approach may be seen as limiting the comparability of our findings with studies that focus exclusively on fabrication, falsification and plagiarism. However, this broader scope allows us to capture a wider range of unethical behaviors, offering a more comprehensive view of the issue. We did not perform an in-depth analysis of the topics addressed by retracted papers, since the main aim was to ascertain the reasons for retraction and their distribution by country. A further limitation may lie in not having carried out a detailed analysis of the journals or language of publication of the retracted papers, though this was not our main aim. Lastly, neither the institutions where these authors worked (e.g., university, hospital, research center) nor the reasons for retractions were analyzed by country, due to the low number of papers retracted.

In conclusion, the principal type of scientific misconduct in LATAM countries consists of ethical and legal problems, especially problems related with authorship (ghost authorship, gift authorship). These results differ from those reported by other studies conducted in different geographic regions and highlight the importance of analyzing scientific misconduct by country or continent. Studies of this nature are necessary in order to ascertain the situation in specific countries and regions, with a view to enabling pertinent institutions to establish mechanisms that could effectively prevent scientific misconduct.

## Data Availability

The datasets generated and/or analyzed during the present study are not publicly available due to [ethical/legal/privacy] restrictions, but are available from the corresponding author upon request.
